# Cyclopamine tartrate, a modulator of hedgehog signaling and mitochondrial respiration, effectively arrests lung tumor growth and progression

**DOI:** 10.1038/s41598-018-38345-1

**Published:** 2019-02-05

**Authors:** Sarada Preeta Kalainayakan, Poorva Ghosh, Sanchareeka Dey, Keely E. Fitzgerald, Sagar Sohoni, Purna Chaitanya Konduri, Massoud Garrossian, Li Liu, Li Zhang

**Affiliations:** 1 Department of Biological Sciences, University of Texas at Dallas, Richardson, TX 75080 USA; 2 Logan Natural Products, Plano, TX 75025 USA; 3 Department of Radiology, The University of Texas Southwestern Medical Center, Dallas, TX 75390-9058 USA

## Abstract

Lung cancer remains the leading cause of cancer-related death, despite the advent of targeted therapies and immunotherapies. Therefore, it is crucial to identify novel molecular features unique to lung tumors. Here, we show that cyclopamine tartrate (CycT) strongly suppresses the growth of subcutaneously implanted non-small cell lung cancer (NSCLC) xenografts and nearly eradicated orthotopically implanted NSCLC xenografts. CycT reduces heme synthesis and degradation in NSCLC cells and suppresses oxygen consumption in purified mitochondria. In orthotopic tumors, CycT decreases the levels of proteins and enzymes crucial for heme synthesis, uptake, and oxidative phosphorylation (OXPHOS). CycT also decreases the levels of two regulators promoting OXPHOS, MYC and MCL1, and effectively alleviates tumor hypoxia. Evidently, CycT acts via multiple modes to suppress OXPHOS. One mode is to directly inhibit mitochondrial respiration/OXPHOS. Another mode is to inhibit heme synthesis and degradation. Both modes appear to be independent of hedgehog signaling. Addition of heme to NSCLC cells partially reverses the effect of CycT on oxygen consumption, proliferation, and tumorigenic functions. Together, our results strongly suggest that CycT suppress tumor growth in the lung by inhibiting heme metabolism and OXPHOS. Targeting heme metabolism and OXPHOS may be an effective strategy to combat lung cancer.

## Introduction

Lung cancer is the leading cause of cancer-related death in the US^1^. About 85–90% of cases are classified as non-small cell lung cancer (NSCLC)^2^. Despite the advent of targeted therapies and immunotherapies, an effective treatment or cure for lung cancer remains an unlikely outcome for most patients. The five-year survival rate remains 10–20%, lower than many other cancers, such as breast (90%) and prostate (99%) cancers^3^. Further, even for early-stage patients typically treated with surgical or radiological procedures, the five-year survival rate is less than 60%, as compared to greater than 95% in the cases of early-stage prostate and breast cancers^4^. Targeted therapies are limited by two factors^5^: Firstly, patients with targetable genomic alterations represent a relatively small percentage of all NSCLC cases. Secondly, resistance to molecularly targeted agents inevitably develops in tumor cells under chronic drug exposure, as further mutations in many potential oncogenic drivers develop. A 2016 study of 17664 patients with NSCLC^6^ showed that the presence of a targetable genetic alteration vs. none was associated with moderately improved first-line progression-free survival (10·0 months vs. 7·1 months; p < 0·0001) and overall survival (16·5 months vs. 11·8 months; p < 0·0001). Recently, immunotherapies have attracted intense interest^7^. Since 2015, the FDA has approved 3 PD-1/PD-L1 checkpoint inhibitors—nivolumab, pembrolizumab, and atezolizumab—for treatment of advanced NSCLC. These inhibitors, compared to docetaxel, generally extend the median overall survival by about 3.0 months. In the front-line setting, the median progression-free survival extended from 6.0 months with platinum-doublet chemotherapy to 10.3 months with pembrolizumab in patients with untreated NSCLC with a high level of PD-L1 expression^8^. As such, for the overwhelming majority of NSCLC patients, immunotherapies and targeted therapies extend survival for only several months^6,8^. Therefore, there is still an urgent need to develop novel therapeutic strategies, by targeting previously under-tested cellular functions and pathways, to substantially improve lung cancer patient survival rates.

Notably, several recent studies showed that the drug-resistant cells of acute and chronic myeloid leukemia, breast cancer, and melanoma depend on OXPHOS and that targeting oxidative metabolism and mitochondrial respiration overcomes their drug resistance^9–13^. Although NSCLC tumors are metabolically heterogeneous, stable isotope resolved-metabolomics for pathway tracing identified a common feature of human NSCLC tumors: pyruvate from elevated glycolysis enters and intensifies the TCA cycle^14^. An intensified TCA cycle should provide more TCA intermediates for biosynthesis and more NADH for ATP generation via OXPHOS. Further, it was shown that lactate fuels the TCA cycle in molecularly heterogeneous tumors^15^. A separate study using two genetically engineered mouse models for lung cancer carrying different genetic mutations (Kras^LSL-G12D/+^Trp53^−/−^ and Kras^LSL-G12D/+^Stk11^−/−^) showed that the contribution of lactate to the TCA cycle is higher than that of glucose^16^. Additionally, components of OXPHOS complexes and markers of mitochondrial biogenesis are found to be highly predictive of reduced overall survival in NSCLC patients^17^.

Recent work in the authors’ lab indicated that cyclopamine tartrate (CycT) inhibits mitochondrial respiration in NSCLC cell lines^18^, but it is unknown whether it can suppress lung tumors *in vivo*. Here, we show that CycT was highly effective at suppressing NSCLC cell tumorigenic functions and NSCLC xenograft tumors in NOD/SCID mice. Furthermore, we show that CycT directly inhibited mitochondrial respiration/OXPHOS using purified mitochondria. CycT reduced heme synthesis and degradation in NSCLC cells. It strongly diminished the levels of proteins involved in heme biosynthesis, uptake, and transport in NSCLC tumors. Heme serves as a prosthetic group in proteins and enzymes involved in oxygen transport, utilization, and storage, such as globins and cytochromes^19^. Multiple subunits in OXPHOS complexes II-IV contain heme. We found that CycT decreased the levels of heme- and non-heme-containing subunits of OXPHOS complexes in lung xenograft tumors. Interestingly, addition of heme at least partially reversed the effects of CycT on oxygen consumption, proliferation, and tumorigenic functions in NSCLC cells. Notably, CycT effectively alleviated tumor hypoxia. These results strongly suggest that CycT suppresses NSCLC tumor growth and alleviates tumor hypoxia by directly inhibiting OXPHOS and by lowering the levels of proteins and enzymes involved in OXPHOS.

## Results

### CycT effectively inhibits cell proliferation and tumorigenic functions in NSCLC cell lines

**Figure 1:**
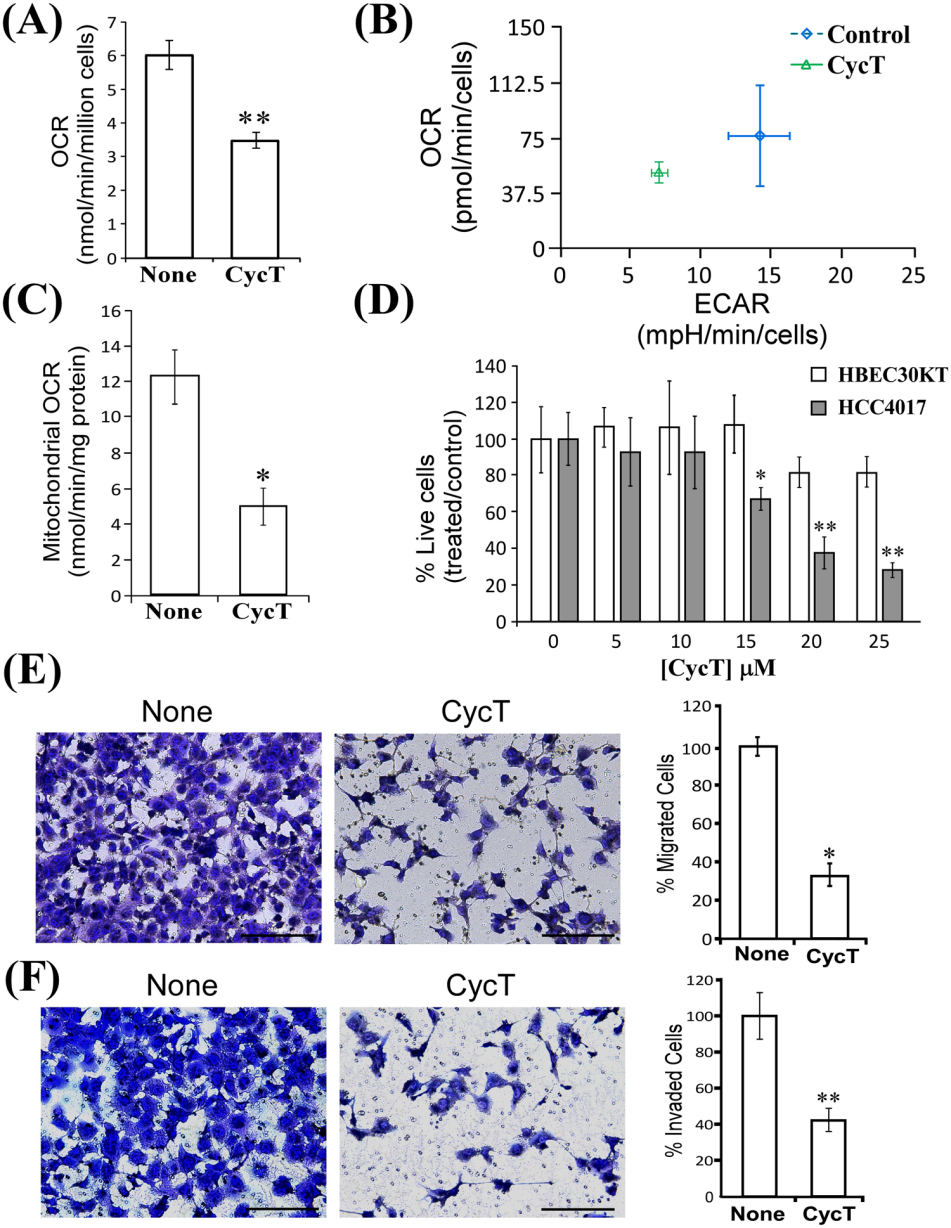
Cyclopamine tartrate (CycT) inhibits proliferation and tumorigenic functions of NSCLC cell lines. (**A**) The effect of CycT on oxygen consumption rates (OCR) in H1299 cells. (**B**) The effects of CycT on extracellular acidification rate (ECAR) in H1299 cells. H1299 cells were incubated with CycT (25 μM) for 3 hours prior to measurements in both (**A**–**C**). CycT directly inhibits oxygen consumption in purified mitochondria from H1299 cells. Purified mitochondria was incubated with CycT (25 μM) for 10 minutes prior to measurements of OCR. (**D**) CycT strongly inhibits cell proliferation of the NSCLC cell line HCC4017, but not the non-tumorigenic HBEC30KT cell line representing normal lung epithelial cells. (**E**) CycT inhibits transwell migration of H1299 cells. (**F**) CycT inhibits invasion of H1299 cells. CycT (25 μM) was incubated with cells for 6 days. Data are plotted as mean ± standard deviation. Scale bar: 200 µm. For statistical analysis, the levels in treated cells were compared to the levels in untreated cells with a Welch 2-sample t-test. *p-value < 0.05; **p-value < 0.005.

Although CycT inhibits oxygen consumption in NSCLC cell lines^18^, it was not clear if it is effective in suppressing tumorigenic functions of NSCLC cells and tumor growth *in vivo*. Here, we first examined the short-term effects of CycT on oxygen consumption rate (OCR) after only 3 hours of treatment with a Clark-type electrode. Figure 1A shows that CycT significantly inhibited oxygen consumption. Using an Agilent XF24 extracellular flux analyzer, we measured the effect of CycT on both OCR and extracellular acidification rate (ECAR) (Fig. 1B). Clearly, both methods detected the strong effect of CycT on OCR (Fig. 1A,1B). Interestingly, CycT also reduced ECAR (Fig. 1B). Because CycT was effective in inhibiting OCR after a short treatment time, we tested if CycT can directly inhibit mitochondrial respiration/OXPHOS. We found that CycT quickly inhibited OCR in purified mitochondria from H1299 cells (Fig. 1C). The dose response of H1299 proliferation to CycT was as expected (Fig. S1A). CycT also strongly inhibited NSCLC A549 cells (Fig. S1B). Notably, CycT did not significantly affect the proliferation of non-tumorigenic HBEC30KT cell line representing normal lung epithelial cells^20^ at the doses that significantly inhibited the proliferation of NSCLC cell line HCC4017 representing cancer cells from the same patient (Fig. 1D). Additionally, we characterized the effect of CycT on the tumorigenic functions in NSCLC cell lines. CycT effectively inhibited transwell migration (Fig. 1E), invasion (Fig. 1F), and colony formation (Fig. S1C) in H1299 cells. Likewise, the tumorigenic functions of A549 NSCLC cells were inhibited by CycT (Fig. S1D–S1F). The data show that CycT inhibits mitochondrial respiration/OXPHOS directly and possesses strong anti-tumorigenic activities against NSCLC cells.

### CycT effectively suppresses the growth of subcutaneous NSCLC tumor xenografts

**Figure 2:**
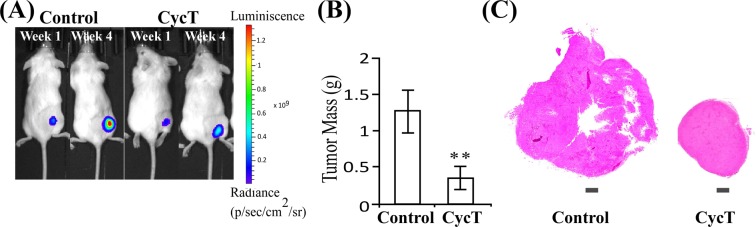
CycT suppresses the growth of subcutaneous NSCLC tumor xenografts. (**A**) Representative bioluminescence images of mice bearing subcutaneous H1299 tumor xenografts treated with CycT (7.5 mg/kg every 3 days, I.V.) or saline (control). n = 6/group. (**B**) Average tumor mass after 4.5 weeks of CycT treatment. Data are plotted as mean ± standard deviation. For statistical analysis, the levels in CycT treated tumors were compared to the levels in control tumors with a Welch 2-sample t-test. **p-value < 0.005. (**C**) Representative H&E images of tumors with CycT or without CycT (control) treatment.

Next, we examined the anti-tumor efficacy of CycT using NOD/SCID mice bearing subcutaneously implanted NSCLC tumor xenografts. Bioluminescence imaging (BLI) showed that CycT significantly delayed tumor growth (Fig. 2A). When mice were sacrificed 6 weeks after initial tumor implantation, the masses of CycT-treated tumors were less than 50% of saline-treated (control) tumors (Fig. 2B). H&E staining also showed that tumor size was substantially reduced by CycT treatment (Fig. 2C). However, lungs are unique organs, and are rich in oxygen. Subcutaneously implanted tumors, which consist of densely packed tumor cells with few stromal cells, may not mimic the tumor microenvironment of human lung tumors.

### CycT is as effective as bevacizumab at suppressing the growth and progression of orthotopically implanted NSCLC tumor xenografts in mice

**Figure 3:**
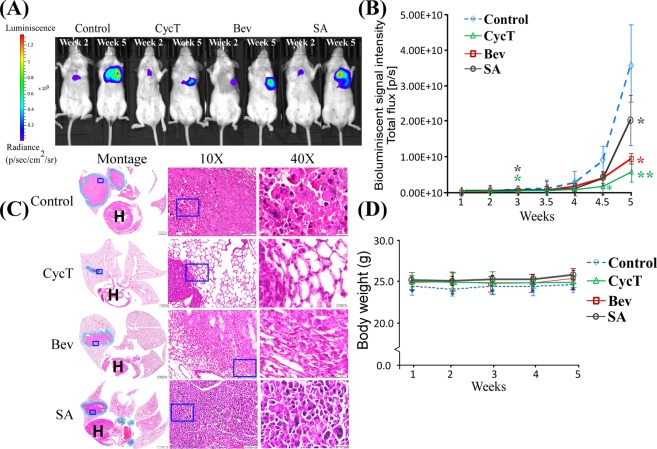
CycT suppresses the growth of NSCLC orthotopic tumor xenografts. (**A**) Representative bioluminescence images of mice bearing orthotopic H1299 tumor xenografts treated with saline (control), cyclopamine tartrate CycT (7.5 mg/kg, I.V.), bevacizumab (Bev, 5 mg/kg, I.P.), and SA (50 mg/kg, I.V.) every 3 days. n = 6/group. (**B**) The quantified luminescence signals representing tumor volumes. Data are plotted as mean ± standard deviation. For statistical analysis, the levels in treated tumors were compared to the levels in control tumors with a Welch 2-sample t-test. *p-value < 0.05; **p-value < 0.005. (**C**) Representative H&E images of control tumors and tumors treated with CycT, Bev or SA. Tumors are marked with light blue outlines. Montage (scale bar: 1 mm), 10X (scale bar: 100 µm), and 40X (scale bar: 20 µm) images of the H&E sections are shown from left to right. The light blue rectangles in Montage and 10X denote the regions shown in 10X and 40X, respectively. (**D**) The body masses of mice under every treatment condition.

Orthotopic tumors have been shown to be more clinically relevant models of lung tumors than subcutaneous xenografts^21,22^. Thus, we decided to test the efficacy of CycT at suppressing orthotopically implanted NSCLC tumor xenografts. We also examined the effect of bevacizumab in comparison. Bevacizumab is an anti-VEGF antibody approved by FDA for treating lung cancer^23^. Bevacizumab may serve as a reference for potential efficacy of CycT in tumor suppression. Inhibition of angiogenesis and inhibition of OXPHOS should both lead to reduction in ATP production in tumor cells via decreasing oxygen supply and consumption, respectively. Thus, CycT and bevacizumab may share overlapping mechanisms in tumor suppression. Additionally, succinyl acetone (SA) is a well characterized inhibitor of the rate-limiting heme synthesis enzyme ALAS1^24^. Its effect in suppressing the proliferation and survival of various cancer cells has been well studied^25–27^. Its effect in animals is also characterized^28,29^. Thus, SA may have some comparative value as well. BLI showed that CycT was very effective at suppressing lung tumor growth and progression and that CycT was as effective as, if not more than, bevacizumab (Fig. 3A,3B). Both CycT and bevacizumab were much more effective than SA. Histological analysis with H&E staining showed that CycT nearly eradicated lung tumors (Fig. 3C). The administration of CycT, bevacizumab, or SA at the indicated doses did not cause strong toxicity in mice, as expected^30^. The body masses of treated mice appeared to be slightly higher than control mice, albeit not statistically significant (Fig. 3D). These results show that CycT has the potential to be a highly effective agent for the treatment of NSCLC.

### CycT decreases the levels of enzymes and proteins involved in heme biosynthesis, uptake, transport, and degradation

To gain insights into the molecular basis underlying the effectiveness of CycT at suppressing lung tumors, we decided to detect the levels of key proteins that CycT may affect. As expected, CycT, not bevacizumab, dramatically decreased the protein levels of Gli1 (see Fig. S2A), a target of SMO and a transcriptional regulator mediating hedgehog (Hh) signaling^31,32^. However, other studies have shown that CycT is not a simple SMO antagonist as it has other functions, including agonist functions^33,34^. Heme is a central molecule for oxidative metabolism and ATP generation via the TCA cycle and OXPHOS. Multiple subunits in OXPHOS complexes II-IV contain heme^19^. Heme also directly regulates many molecular and cellular processes involved in oxygen utilization^35^. Therefore, it would be insightful to examine the effects of CycT on the levels of proteins controlling the levels and flux of heme in tumor cells. However, it is difficult to quantify the levels of proteins in tumor cells of orthotopic lung tumor xenografts using Western blotting, particularly in treated tumors, which are small. Furthermore, changes in mitochondrial proteins, including many hemoproteins, are difficult to detect using proteomic methods, particularly in complex tumor tissues with stromal cells. The difficulty in isolating a pure population of tumor cells from mouse lungs would also make it difficult to gain consistent data from genomic studies, and the correlation between mRNA and protein levels are known to be poor^36^. Thus, we decided to carry out quantitative immunohistochemistry (IHC) analyses.

**Figure 4:**
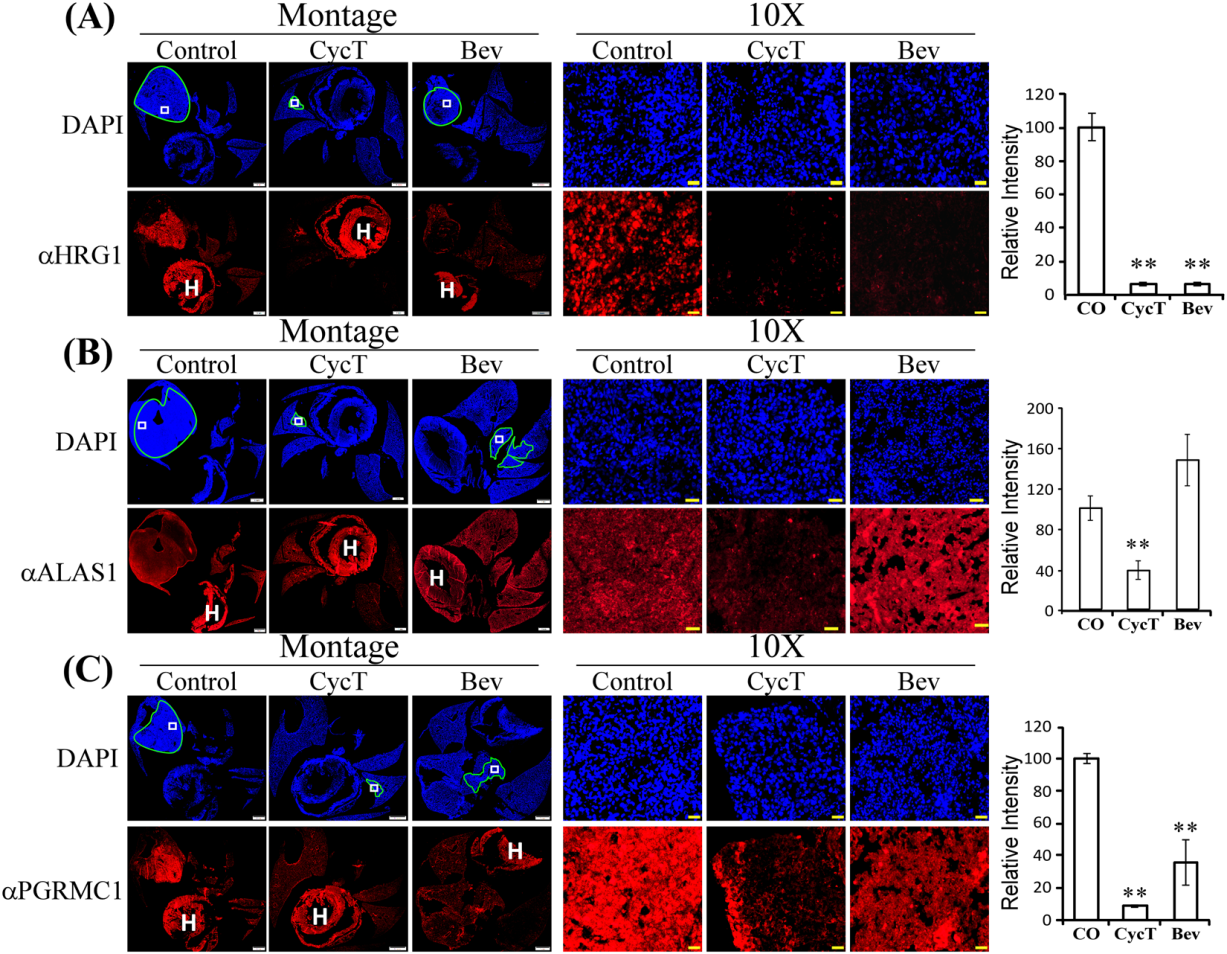
CycT effectively inhibits the levels of proteins and enzymes involved in heme uptake, synthesis, and transport. (**A**) Representative IHC images of H1299 NSCLC tumor tissue sections and graph showing the levels of HRG1 in control and treated tumors. Shown are montages and 10X images of control, CycT-treated, and Bev-treated tumor tissue sections stained with DAPI or antibodies against the indicated protein HRG1. The light blue lines in DAPI images outline the tumors in the lung. The white rectangles in DAPI images denote the regions shown in 10X images. The lines and rectangles are not placed on IHC fluorescent images to avoid the obstruction of tumor images. The heart was often stained and is marked with “H”. Scale bar: montage, 1 mm; 10X, 20 μm. Protein levels were quantified with cellSens dimension software (Olympus), as described in Methods. Data are plotted as mean ± SEM. For statistical analysis, the levels in treated tumors were compared to the levels in control tumors with a Welch 2-sample t-test. **p-value < 0.005. (**B**) The effects of CycT and Bev on the levels of the rate-limiting heme biosynthetic enzyme ALAS1 in orthotopic tumor xenografts. (**C**) The effects of CycT and Bev on the levels of the heme chaperone and sensor protein PGRMC1 in orthotopic tumor xenografts. IHC images are representative of 3 independent experiments.

Figure 4A shows representative images from IHC analyses. The first two panels represent the montages of DAPI (staining nuclei) and fluorescent stains of the detected protein, i.e., the heme transporter HRG1 (SLC48A1). HRG1 is a major heme transporter and is located on endosomes, lysosomes, and cell membranes^37,38^. Notably, the montages show that HRG1 levels were generally much higher in tumor regions than in adjacent normal lung regions (Fig. 4A), indicating its pro-tumorigenic role in lung tumors. Clearly, HRG1 protein levels in NSCLC tumors were dramatically diminished by both CycT and bevacizumab.

**Figure 5:**
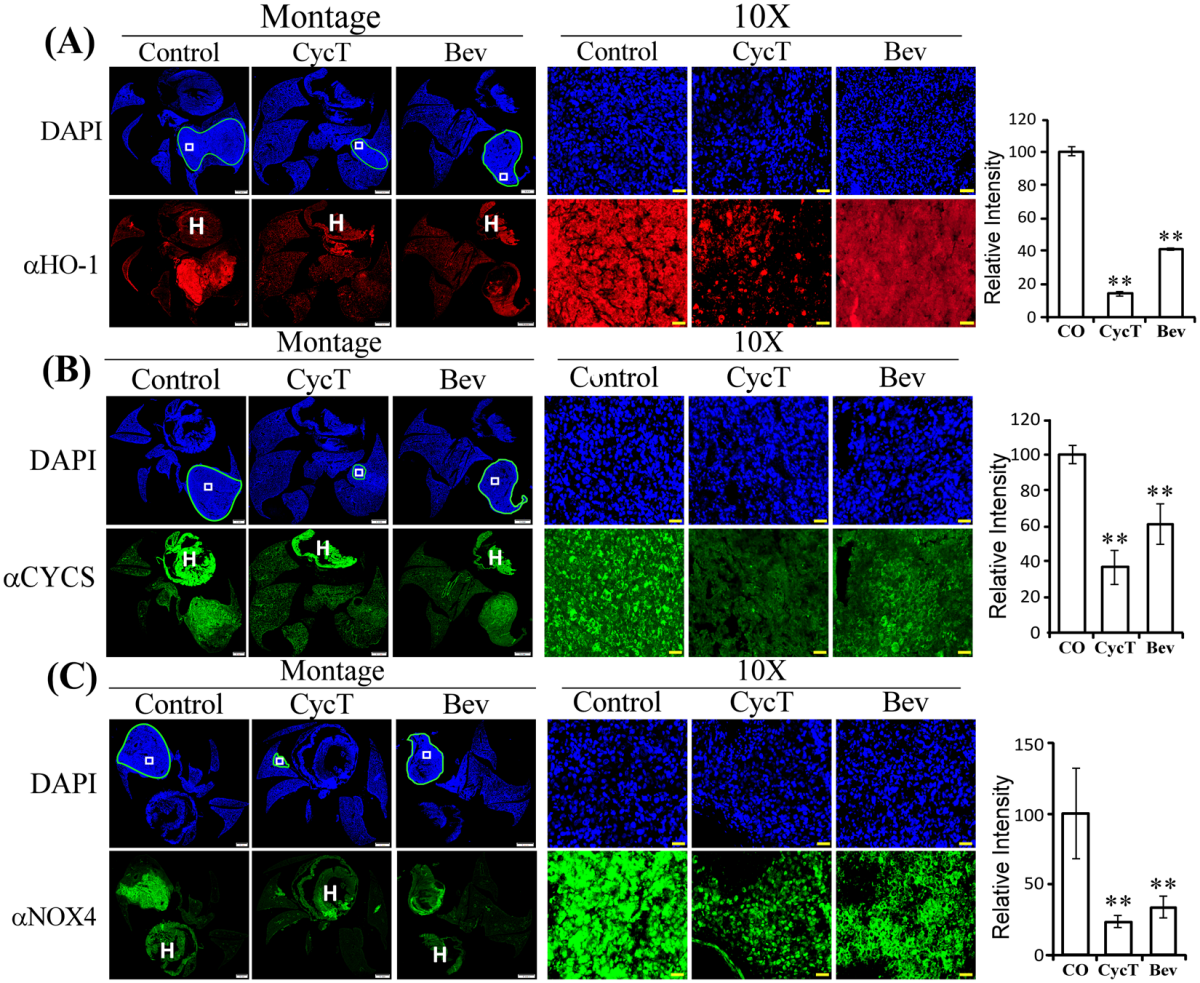
CycT effectively inhibits the levels of enzymes involved in heme degradation, OXPHOS, and pro-tumorigenic hemoproteins. (**A**) The effects of CycT and Bev on the levels of the heme degradation enzyme HO-1 in orthotopic tumor xenografts in control and treated tumors. Shown are montages and 10X images of control, CycT-treated, and Bev-treated tumor tissue sections stained with DAPI or antibodies against the indicated protein HO-1. The light blue lines in DAPI images outline the tumors in the lung. The white rectangles in DAPI images denote the regions shown in 10X images. The lines and rectangles are not placed on IHC fluorescent images to avoid the obstruction of tumor images. The heart was often stained and is marked with “H”. Scale bar: montage, 1 mm; 10X, 20 μm. Protein levels were quantified with cellSens dimension software (Olympus), as described in Methods. Data are plotted as mean ± SEM. For statistical analysis, the levels in treated tumors were compared to the levels in control tumors with a Welch 2-sample t-test. **p-value < 0.005. (**B**) The effects of CycT and Bev on the levels of cytochrome c (CYCS) in orthotopic tumor xenografts. (**C**) The effects of CycT and Bev on the levels of NOX4 in orthotopic tumor xenografts. IHC images are representative of 3 independent experiments.

Another heme transporter on the cell membrane, HCP1 (SLC46A1), was also significantly reduced by CycT in orthotopic tumors (Fig. S2B). The levels of the rate-limiting heme synthetic enzyme ALAS1 were reduced by CycT, but not by bevacizumab (Fig. 4B). We also detected the level of a putative heme sensor and heme chaperone necessary for maintenance of cellular heme and hemoprotein levels, PGRMC1^39^. The levels of PGRMC1 were greatly reduced by CycT, while bevacizumab reduced them to a lesser extent (Fig. 4C). Likewise, another putative heme sensor and heme chaperone protein, GAPDH^40^, was greatly reduced by CycT (Fig. S2C). Interestingly, the levels of the heme degradation enzyme HO-1 were reduced by CycT, as well as bevacizumab (Fig. 5A). These results show that important proteins involved in heme uptake, biosynthesis, and maintenance are strongly reduced by CycT, while bevacizumab has lesser effects.

### CycT effectively reduces the levels of subunits of OXPHOS complexes and other oxygen-utilizing hemoproteins

A coordinated strong reduction in heme biosynthesis and uptake should limit the availability of cellular heme for producing hemoproteins, as well as non-heme proteins, due to the role of heme in coordinating the expression of all proteins involved in oxygen utilization, such as OXPHOS. To test this idea, we detected the levels of several subunits of OXPHOS complexes in treated and control H1299 NSCLC tumor xenografts. Indeed, CycT significantly reduced the levels of cytochrome c (CYCS, acting between OXPHOS Complex III and IV, Fig. 5B), UQCRC2 (a subunit of OXPHOS Complex III, Fig. S3A), and ATP5F1B (a subunit of OXPHOS Complex V, Fig. S3B). Further, CycT strongly decreased the levels of pro-tumorigenic hemoproteins, cyclooxygenase-2 (PTGS2) and cytochrome P450 (CYP1B1) in H1299 tumors, while bevacizumab did not affect PTGS2 (Fig. S3C,S3D). Interestingly, another heme-containing, ROS-producing NADPH oxidase, NOX4, was reduced by CycT and bevacizumab in H1299 tumors (Fig. 5C). NOX4 promotes angiogenesis and is tumorigenic^41^. Together, these results show that CycT is highly effective in reducing the levels of key tumorigenic proteins required for oxygen metabolism and ATP generation.

**Figure 6:**
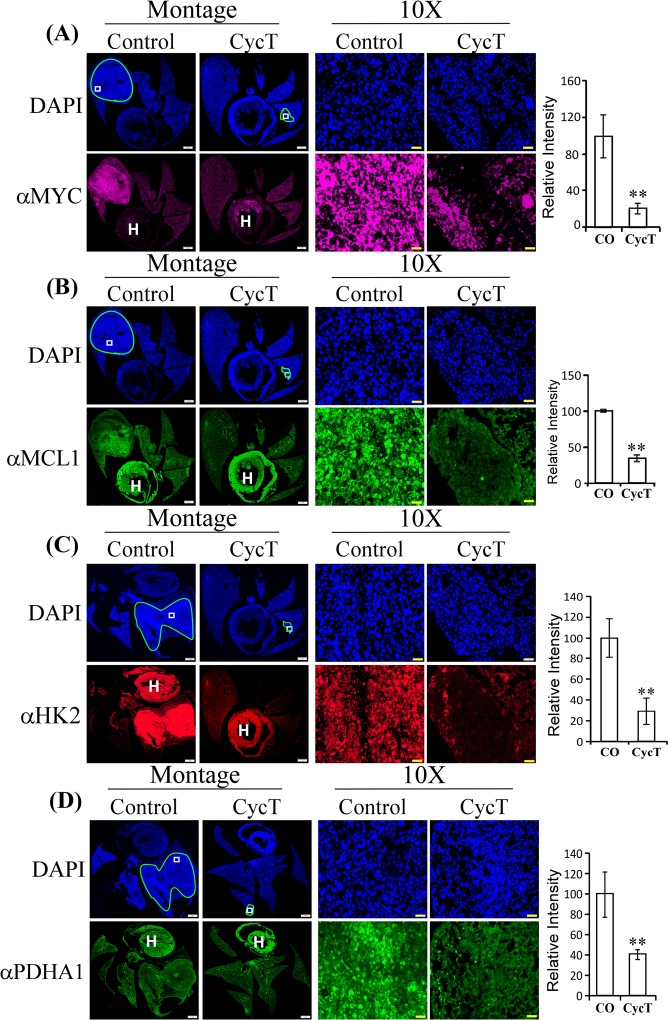
CycT diminishes levels of OXPHOS regulators and glycolytic enzymes. The effects of CycT on the levels of OXPHOS-promoting regulators MYC (**A**) and MCL1 (**B**) and on the levels of hexokinase II (**C**) and pyruvate dehydrogenase (**D**) in NSCLC tumors. Data are plotted as mean ± SEM. For statistical analysis, the levels in treated tumors were compared to the levels in control tumors with a Welch 2-sample t-test. **p-value < 0.005. HK2: hexokinase II; PDHA1: pyruvate dehydrogenase. IHC images are representative of 3 independent experiments.

Furthermore, we examined the effect of CycT on the levels of two previously identified regulators promoting OXPHOS, MYC and MCL1. Lee *et al*. showed that MYC and MCL1 cooperate to promote resistance to chemotherapy in breast cancer stem cells by increasing OXPHOS^13^. Likewise, it appeared that the levels of MYC and MCL1 were much higher in NSCLC tumor cells relative to adjacent normal lung cells, and CycT strongly decreased their levels in tumor cells (see Fig. 6A,6B). These results coincide with the effects of CycT on OXPHOS and enzyme subunits and suggest that one way by which CycT inhibits OXPHOS is by acting on MYC and MCL1.

### CycT reduces heme synthesis and degradation, and addition of heme partially reverses the effect of CycT on oxygen consumption, proliferation, and tumorigenic functions in NSCLC cells

**Figure 7:**
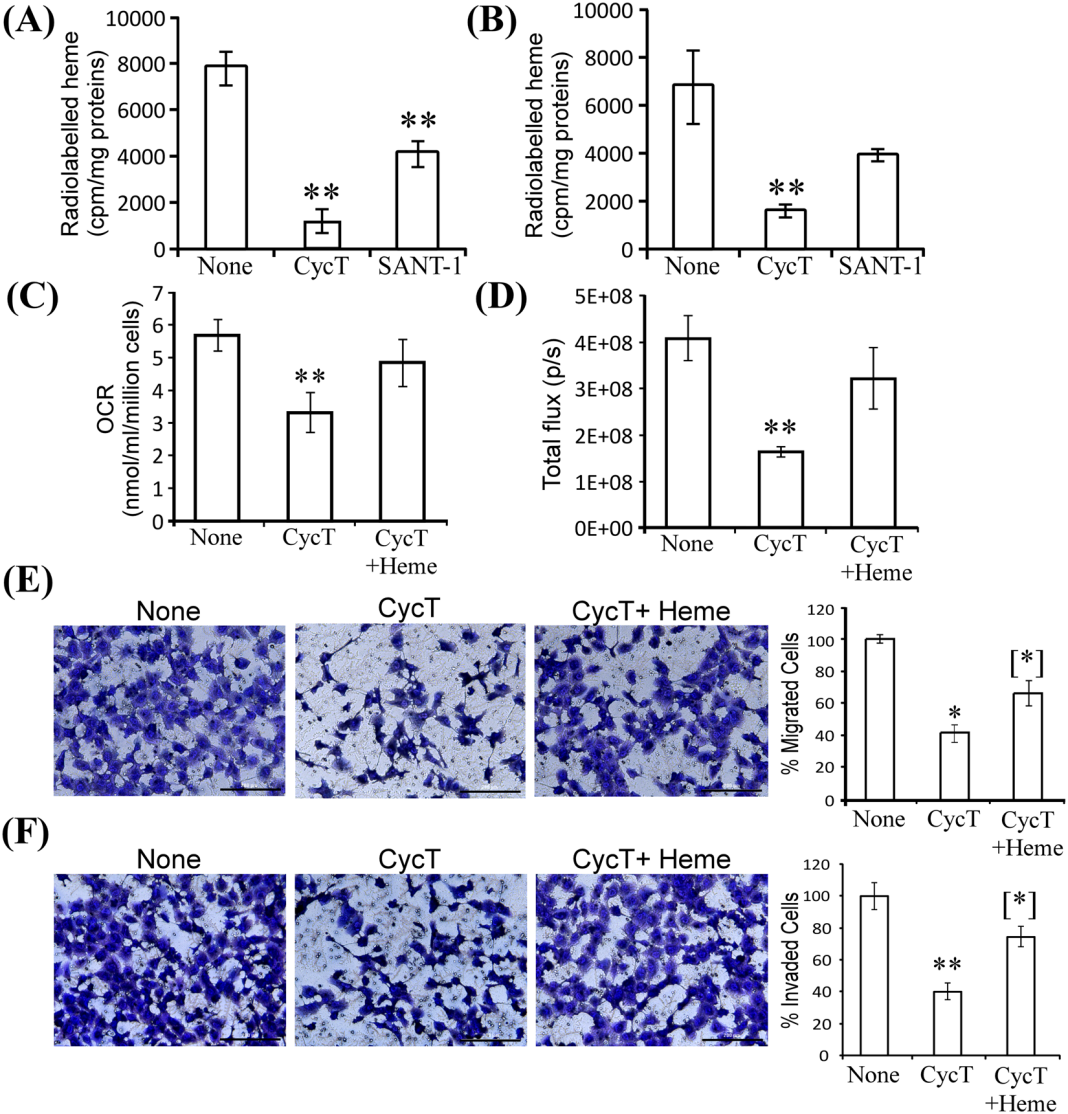
CycT diminishes heme biosynthesis and degradation, and addition of heme partially reverses the effects of CycT on OCR, cell proliferation, and tumorigenic functions in NSCLC cells. CycT, not the SMO antagonist SANT-1, strongly diminishes heme biosynthesis (**A**) and degradation (**B**) in H1299 NSCLC cells. 25 μM CycT and 50 μM SANT-1 were used. Data are plotted as mean ± standard deviation. (**C**) Addition of heme (10 μM) partially reverses the effect of CycT on oxygen consumption rate (OCR) in H1299 cells. (**D**) Addition of heme (10 μM) partially reverses the effect of CycT on the proliferation rate of H1299 cells. (**E**) Addition of heme (10 μM) partially reverses the effect of CycT on migration capability in H1299 cells. (**F**) Addition of heme (10 μM) partially reverses the effect of CycT on invasion capability in H1299 cells. For statistical analysis, the levels in CycT-treated cells were compared to the levels in untreated cells, and the levels in cells with heme added back were compared to the levels in CycT-treated cells. P-values were calculated with a Welch 2-sample t-test. *p-value < 0.05, **p-value < 0.005 for the difference between CycT-treated and untreated cells; [*] p-value < 0.05 for difference between CycT-treated cells and CycT-treated cells with heme added back.

To further ascertain whether CycT affects heme metabolism via its SMO-antagonist activity, we compared the effects of CycT and the strong antagonist SANT-1 on heme synthesis and degradation. Figure 7A shows that CycT reduced the levels of heme synthesis in H1299 cells more than 6-fold, while SANT-1 reduced it less than 2-fold. Likewise, CycT reduced the levels of heme degradation about 4-fold, while the effect of SANT-1 was not significant (Fig. 7B). Further, we found that addition of heme significantly reversed the reduction of CycT on OCR (Fig. 7C) and proliferation (Fig. 7D) in H1299 NSCLC cells. Heme addition also partially reversed the effect of CycT on NSCLC cell tumorigenic functions (Fig. 7E,7F). Together, these results in Figs 4–7 strongly suggest that CycT reduced heme synthesis and degradation and the levels of oxygen utilizing hemoproteins and that this effect at least in part accounts for the anti-tumor effect of CycT in the lung.

### CycT decreases the levels of proteins and enzymes involved in glucose consumption

It is worth noting that CycT strongly inhibited ECAR (Fig. 1B). To further verify this, we examined the effect of CycT on the levels of proteins and enzymes involved in glucose consumption and glycolysis. Figure 6C shows that CycT strongly reduced the levels of the first enzyme in glycolysis, hexokinase II (HK2), the main hexokinase in lung cancer cells. Likewise, CycT also strongly reduced the levels of pyruvate dehydrogenase (PDHA1) (Fig. 6D) and glucose transporter SLC2A1 (GLUT1, Fig. S3E) in tumor cells. The effects of CycT on enzymes involved in glucose consumption and glycolysis are in accord with its effect on ECAR (Fig. 1B). To ensure that our quantitation is not biased, we show that the levels of an array of diverse cellular proteins, including structural proteins β-actin, filamin FLNA, ER marker KDEL (KDELC1), signaling and transcriptional proteins ABL1, KDMC4, SMARCC2, and EFNA2, as well as heat shock protein Hsp27 (Fig. S4A–S4H), are not changed significantly by CycT in lung tumors.

It is worth noting that heme synthesis requires iron. However, heme synthesis in non-erythroid cells is known to be unaffected by iron^42^. Iron deficiency causes insufficient heme synthesis in erythroid cells, leading to iron deficient anemia. It is likely that iron levels in live mammals does not become low enough to affect synthesis in nonerythorid cells. Nonetheless, we detected the levels of transferrin receptor (TFRC), which is responsible for cellular iron uptake from the circulation^43^, and ferroportin (SLC40A1), which is responsible for cellular iron export to the blood^44^. We found that TFRC levels were not changed by CycT (Fig. S5A), while ferroportin levels were reduced by less than 2-fold (Fig. S5B). The data suggest that intracellular iron availability is unlikely to be affected by CycT treatment in tumors.

### CycT alleviates hypoxia in orthotopic xenograft lung tumors

**Figure 8:**
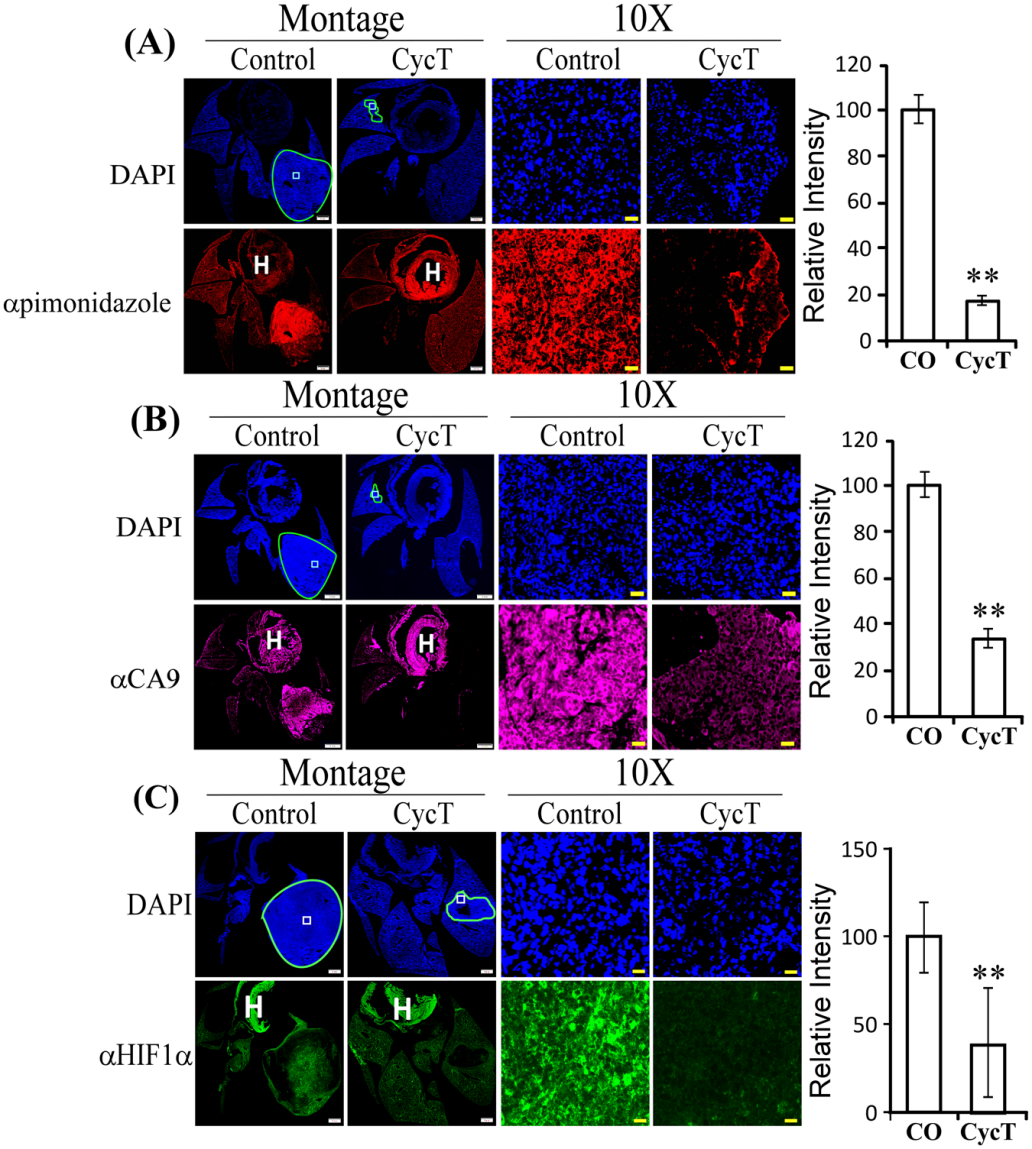
CycT alleviates tumor hypoxia. The effects of CycT on the levels of exogenous hypoxia-marker pimonidazole labeling (**A**), the levels of hypoxia-inducible CA9 enzyme (**B**), and the levels of hypoxia-inducible factor HIF1α (**C**) in orthotopic tumor xenografts. Scale bar: Montage, 1 mm; 10X, 20 μm. Data are plotted as mean ± SEM. For statistical analysis, the levels in treated tumors were compared to the levels in control tumors with a Welch 2-sample t-test. **p-value < 0.005. IHC images are representative of 3 independent experiments.

Tumor hypoxia promotes several processes critical for cancer progression, including angiogenesis, epithelial-mesenchymal transition (EMT), migration/invasion, metastasis, immune surveillance, and resistance to chemotherapy and radiotherapy^45–48^. It is an independent marker of poor prognosis in many types of human cancer^49,50^. Particularly, substantial tumor hypoxia exists in NSCLC, even in early-stage tumors^51^. Targeting hypoxia is crucial for improving therapeutic outcome for NSCLC^52^. Therefore, we examined hypoxia in NSCLC tumors and the effect of CycT on tumor hypoxia. To this end, we used endogenous hypoxia marker Carbonic Anhydrase 9 (CA9) and exogenous hypoxia marker pimonidazole^53,54^. Notably, in the control lungs, both pimonidazole labeling and CA9 protein showed higher intensities in tumor regions relative to normal lung regions, indicating the existence of tumor hypoxia (see the control samples in Fig. 8A,8B). In contrast, the levels of pimonidazole labeling and CA9 protein are both significantly reduced in CycT-treated cells. Likewise, the levels of the hypoxia-inducible factor HIF1α were also significantly reduced in CycT-treated cells (Fig. 8C), indicating reduced hypoxia. These results show that the NSCLC tumors were hypoxic and CycT reduced tumor hypoxia consistently.

Previously, we showed that CycT causes apoptosis in NSCLC cells^18^. In orthotopic NSCLC tumors, we did not detect signs of apoptosis in untreated tumors (Fig. S5C) or in CycT-treated small tumors (Fig. S5D). However, we detected signs of apoptosis in CycT-treated tumors with a slightly larger size (Fig. S5E). These results suggest that CycT causes cell death in lung tumors and that debris from apoptotic cells are cleared in the lung as the treatment progresses. This conclusion is consistent with our previous results using NSCLC cell lines^18^. Together, these results show that CycT can suppress cell proliferation and apoptosis in NSCLC cells.

## Discussion

The latest experimental evidence from studies of human NSCLC patients has shown that NSCLC tumor cells exhibit high levels of glucose oxidation and lactate consumption^14,15^. Evidently, elevated glucose consumption and glycolysis in tumor cells do not necessarily lead to diminished oxidative metabolism and OXPHOS. In fact, elevated glucose consumption in human NSCLC tumors are coupled to intensified glucose oxidation, TCA cycle, and lactate utilization^14,15^. Numerous previous studies have shown that high glycolytic rates occur concomitantly with high OXPHOS rates in cells of most tumors and that function of mitochondrial OXPHOS is intact in most tumors (for a review, see)^55^. Several studies have unequivocally demonstrated the importance of mitochondrial OXPHOS in many tumors. Viale *et al*. showed that a sub-population of dormant tumor cells surviving oncogene ablation, which are responsible for tumor relapse, rely on OXPHOS for survival^56^. LeBleu *et al*. showed that migratory and invasive cancer cells favor mitochondrial respiration and increased ATP production^57^. Tan *et al*. showed that tumor cells without mitochondrial DNA (mtDNA) exhibit delayed tumor growth and that tumor formation is associated with the acquisition of mtDNA from host cells^58^. Importantly, several studies demonstrated that oxidative metabolism and OXPHOS are crucial for conferring drug resistance in cancer cells and cancer stem cells. Farge *et al*. showed that OXPHOS contributes to acute myeloid leukemia resistance to cytarabine and that targeting mitochondrial metabolism induces an energetic shift toward low OXPHOS and strongly enhanced anti-leukemic effects of cytarabine^9^. Kuntz *et al*. showed that targeting mitochondrial OXPHOS eradicates drug-resistant chronic myeloid leukemia stem cells^10^. Lee *et al*. showed that MYC and MCL1 confer chemotherapy resistance by increasing mitochondrial OXPHOS in triple negative breast cancer stem cells^13^. Interestingly, we have shown that viable NSCLC tumor cells resistant to the vascular disrupting agent combretastatin A-4 phosphate exhibit further elevated levels of proteins/enzymes relating to heme metabolism and function^59^. Clearly, NSCLC cells and drug-resistant cells or stem cells of many cancers require mitochondrial OXPHOS.

Heme is a central factor in mitochondrial respiration and oxygen metabolism^60^. It is critical for the biogenesis of OXPHOS complexes II-IV^61^. Furthermore, heme serves as a signaling molecule that directly regulates diverse processes ranging from gene transcription to potassium channel activation^62,63^. Recent experimental data from other studies also strongly supported the idea that mitochondrial respiration and heme function are crucial for lung tumorigenicity. For example, Sotgia and Lisanti identified >180 mitochondrial gene probes, including components of the OXPHOS complexes, that effectively predicted significantly reduced overall survival in NSCLC patients^64^. Another genome-wide expression study in 49 tumors and 42 non-involved fresh-frozen lung tissues of 64 adenocarcinoma patients identified 232 annotated, differentially-expressed genes, 63 of which (p-value < 0.001) are involved in heme binding, absorption, transport, and Wnt signaling^65^. Additionally, epidemiological studies indicated a positive association between intake of heme from meat and lung cancer^66^. Clearly, heme is a unique pro-tumorigenic molecule with both metabolic and signaling functions. Likewise, oxygen-utilizing hemoproteins, such as OXPHOS complexes, are also pro-tumorigenic. Thus, shutting down heme synthesis, heme uptake, and the expression of hemoproteins can be a viable strategy for effective suppression of lung tumorigenesis and for overcoming drug resistance.

CycT is a more potent, water-soluble form of cyclopamine^30^. Cyclopamine was initially identified as an inhibitor of smoothened (SMO), a G protein-coupled receptor that positively regulates hedgehog (Hh) signaling^32^. Since then, an array of SMO antagonists have been developed and tested for cancer treatment^67,68^. Vismodegib (GDC0449, Curis/Roche) was approved in 2012 by the US FDA for treating locally advanced and metastatic basal cell carcinoma^69^. However, it is not effective against other cancers^70,71^. Importantly, cocrystal structures of SMO with several small molecules showed that the binding site for cyclopamine is distinct from the sites for other antagonists^34^. Evidently, the mode by which cyclopamine binds to SMO is more similar to that of SMO agonist SAG1.5 than other antagonists. Indeed, cyclopamine is a partial agonist capable of concomitant inhibition of canonical and activation of non-canonical hedgehog signaling^33^. Therefore, it is very likely that CycT has distinct anti-cancer activities different from other SMO antagonists.

Indeed, our data show that CycT has anti-cancer activities that is independent of Hh signaling. Firstly, the quick inhibition of CycT on oxygen consumption in purified mitochondria (Fig. 1C) show that CycT acted directly on OXPHOS independently of Hh signaling. Secondly, CycT exerted a much stronger effect on heme synthesis and degradation than the SMO antagonist SANT-1 (Fig. 7A,7B). Notably, addition of heme partially reversed the effect of CycT on oxygen consumption and cancer cell proliferation and tumorigenic functions (Fig. 7C–7F). The result supports the idea that CycT suppresses tumorigenic functions and tumor growth by lowering heme metabolism and OXPHOS. This idea is further supported by the observation that CycT effectively diminished the levels of proteins involved in heme biosynthesis, uptake, and transport (Figs. 4 and 5). CycT also diminished levels of subunits of OXPHOS complexes and other hemoproteins with pro-tumorigenic functions, including PTGS2, CYP1B1, and NOX4 (Figs. 5C and S3C,S3D). Furthermore, CycT strongly reduces the levels of MYC and MCL1 in NSCLC tumors. MYC and MCL1 promote OXPHOS in breast cancer stem cells^13^. They likely have the same function in NSCLC cells. The reduced levels of these regulators are in agreement with reduced levels of OXPHOS proteins in CycT-treated tumors.

Together, the data show that CycT exerts multiple effects on the pathways of ATP generation in NSCLC tumors. Firstly, CycT acts quickly to inhibit OCR in purified mitochondria and in NSCLC cells. Secondly, CycT decreases heme synthesis and degradation. Thirdly, CycT reduces the levels of OXPHOS-promoting regulators MYC and MCL1. Fourthly, CycT strongly decreases the levels of heme-related proteins/enzymes and OXPHOS complex subunits in NSCLC tumors. Lastly, CycT reduces the levels of glycolytic enzymes and the major glucose transporter SLC2A1 (GLUT1). These multiple effects of CycT on OXPHOS and glucose oxidation make it a uniquely effective agent to suppress ATP generation in NSCLC tumors.

The multiple effects of CycT on metabolic and signaling processes are not unusual. Like CycT, metformin is a small molecule isolated from plants. Metformin has been historically used to treat Type II diabetes. Since 2001, numerous studies have shown that metformin has anticancer activities in mammals^72–77^. At the molecular level, metformin reduces mitochondrial respiration^78^, disrupts lipid metabolism, glucose metabolism, tricarboxylic acid cycle, the methionine cycle, the folate cycle, as well as nucleotide synthesis^79,80^, and inhibits TOR signaling^81,82^.

It is also worth noting that the effects of CycT on NSCLC cells *in vitro* may differ from its effects on NSCLC tumors *in vivo*. Stromal cells in the tumor microenvironment likely alter the molecular effects of CycT on tumor cells. For example, while our previous studies in NSCLC cells did not detect an effect of CycT on HO-1 levels^18^, figure 5A shows that HO-1 levels were reduced in CycT-treated lung tumors. The detected effects of CycT on lung tumors are likely a result of complex interactions among drug, tumor cells, and stromal cells. Nonetheless, our *in vitro* and *in vivo* data are all consistent in that CycT inhibits OXPHOS, heme synthesis and degradation, and reduces Gli1 levels *in vitro*^18^ and *in vivo* (Figs 1, 4–7, S2 and S3).

The toxicological profile of CycT has been previously characterized, and it is well tolerated by humans and mice^30^. CycT and cyclopamine do not appear to affect red cells^30,83^. Heme synthesis rates in erythroid cells are much higher those in non-erythroid cells and is developmentally regulated^42^. Factors that affect heme synthesis in non-erythroid cells generally do not affect erythroid cells. The blood concentration of cyclopamine after infusion at a high dose of 160 mg/kg/day is about 2 μM^83^. This concentration of cyclopamine or CycT does not affect hematopoietic differentiation in mice. Thus, it is conceivable that a relatively low dose (7.5 mg/kg every 3 days) used to suppress lung tumors here does not affect blood cells. Interestingly, bevacizumab is not an inhibitor of Hh signaling; it does not affect Gli1 levels, as expected (Fig. S2A). However, it reduces the levels of certain proteins involved in heme metabolism and OXPHOS, such as HRG1, HCP1, PGRMC1, Cytochrome c, UQCRC2, ATP5F1B, CYP1B1, and NOX4, albeit to lesser extents than CycT. These results are consistent with the idea that the efficacy of CycT at suppressing mitochondrial and heme functions are not attributable to its function as a SMO antagonist. The overlapping effects of CycT and bevacizumab on these proteins relating to OXPHOS are consistent with their roles on reducing ATP generation via OXPHOS in tumor cells.

In summary, while CycT suppresses Hh signaling *in vitro* and *in vivo*, as shown by the decrease of Gli1 levels^18^ (Fig. S2A), our data here show that CycT can act independently of Hh signaling to inhibit OXPHOS, heme synthesis and degradation. CycT acts via multiple modes to diminish OXPHOS in lung tumors. Targeting OXPHOS is an effective strategy to overcome drug resistance of other cancers, such as leukemia and triple-negative breast cancer^9,10,13^. Our data showing the multiple molecular effects of CycT on lung tumors provides a viable therapeutic strategy to combat lung cancer and other drug-resistant cancers.

## Methods

### Reagents, cell culture, measurements of OCR and ECAR, and analyses of tumorigenic functions

Cyclopamine tartrate (>99% purity) was provided by Logan Natural Products. D-Luciferin and the Opal 4 color IHC kit were purchased from PerkinElmer (USA). Bevacizumab (17.26 mg/ml) was provided by Genentech, Inc. SANT-1 and Pimonidazole Hydrochloride were purchased from Santa Cruz Biotechnology and Hypoxyprobe, Inc., respectively. [4-^14^C]-5-aminolevulinic acid was custom synthesized by PerkinElmer. NSCLC cell lines H1299 (CRL-5803) and A549 (CRM-CCL-185) were purchased from American Type Culture Collection (ATCC). Cell lines expressing luciferase were generated by infection with lentiviral particles bearing the EF1a-Luciferase gene (AMSBIO) at passage 3. Cell lines were authenticated by short tandem repeat (STR) profiling (PowerPlex 16HS) (Genetica DNA Laboratories, Inc.) and were found to be 96% identical to the standard (authentication requires > 80%). The TumorTACS^TM^ *In Situ* Apoptosis Detection Kit was purchased from Trevigen, Inc.

Oxygen consumption was measured with a Clark-type electrode, as described previously^84^. Purified mitochondria were prepared from H1299 cells as described^85^. OCR was measured in the presence of OXPHOS substrates, with a Clark-type electrode and normalized with protein amounts as described^85^. To measure OCR and ECAR with a Seahorse Bioscience XF243 Extracellular Flux Analyzer, 2500 cells were seeded in the Seahorse XF Cell Culture Microplate for 3 days, and then the Seahorse Bioscience XF Cell Mito Stress Test Assay Kit was used. Cell proliferation was measured by detecting luciferase activity. Cell migration and invasion assays were carried out with BD Falcon cell culture inserts (Corning Life Sciences) following the manufacturer’s protocols. For the colony formation assay, 5000 NSCLC cells were seeded per well in 6-well tissue culture plates in triplicates. Cells were treated with 25 μM CycT for 6 days. Cells were then fixed and stained with 0.5% crystal violet. Images were acquired by using the Carestream Gel Logic GL-112 imaging system.

### Measurement of heme synthesis and degradation

Measurement of heme synthesis in cells was carried out in triplicates exactly as described^86^. Briefly, cells were treated with or without 25 μM CycT or 50 μM SANT-1 for 7 days, 0.3 μCi [4-^14^C]-5-aminolevulinic acid (ALA) was the added to each well for 15 hours. Heme was subsequently extracted, and radiolabeled heme was quantified exactly as described^86^. The levels of heme degradation were calculated by subtracting the amounts of radiolabeled heme in cells grown for another 24 hours from the amounts of radiolabeled heme in cells after incubation with 4-^14^C-5-aminolevulinic acid (ALA) for 15 hours.

### Animals

NOD/SCID (CRL:394) mice were purchased from Charles River, maintained in a pathogen-free facility in accordance with the Protocol # 13-05 approved by IACUC of UT Dallas.

### Subcutaneous and orthotopic xenograft mouse models

For subcutaneous models, 2.5 × 10^6^ H1299-Luc cells in serum-free medium containing 50% Matrigel were injected subcutaneously into the left flank region of 4–6 weeks old female NOD/SCID mice (n = 6 per group). Mice were randomized into two groups that received I.V. saline (for control) and cyclopamine tartrate (CycT, 7.5 mg/kg every 3 days), respectively. Body masses were recorded once every week. When the tumors reached 1 cm^3^, mice were euthanized by cervical dislocation. Tumors were resected and weighed. This experiment was repeated two times.

For orthotopic models, 0.75 × 10^6^ H1299-luc cells (passages 3–5) in serum-free medium containing 50% Matrigel were implanted orthotopically in 6–8-week-old female NOD/SCID mice. Mice were anesthetized and placed in right lateral decubitus position. H1299-luc cells were injected about 1.5 cm above the lower left rib line through the intercostal region. Mice were observed until they revived from anesthesia. Mice were randomized into four groups (n = 6 per group) that received I.V. saline (for control), CycT (7.5 mg/kg, I.V.), bevacizumab (5 mg/kg, I.P.), and SA (50 mg/kg, I.V.) every 3 days. Treatments started 4 days after cell implantation. This experiment was repeated three times. The dose and route of administration for bevacizumab were recommended by Genentech, Inc. The recommendation is based on extensive previous studies, including those showing that the different routes of administration by I.V. or I.P. do not affect therapeutic efficacy of bevacizumab^87–89^. A moderate dose of SA was chosen based on previous studies of its effect on animals^28,29^.

### *In vivo* bioluminescence imaging (BLI)

Mice bearing subcutaneous or orthotopic tumor xenografts were imaged with an IVIS Lumina III *In Vivo* Imaging system. Mice were anesthetized with 2% isoflurane. Luciferin (80 µl of 40 mg/ml) was administered subcutaneously. A BLI time course was acquired over 30 mins (Exposure time: auto, F Stop: 1.2, Binning: medium). The images were quantified using Living Image software version 4.5.2 (Perkin Elmer). Regions of interest (ROIs) were selected. Bioluminescence signals between 600 to 60000 counts were accepted as authentic signals. The total bioluminescent signals (photon/sec) from ROIs of mice were calculated according to the manufacturer’s instructions. Analyses of BLI data were done by personnel who were blinded to the objectives of the study. BLI data of the mice with tumors outside the lungs were excluded.

### Tissue processing and hematoxylin and eosin (H&E) staining

60 mg/kg pimonidazole-HCl was administered to mice via tail vein 90 minutes before sacrifice. Lungs were removed and fixed in 4% formalin. Paraffin embedding was performed at the histopathology core at UTSW Medical Center. The paraffin blocks were sectioned into 5 µm sections which were utilized for H&E staining and immunohistochemical staining.

### Immunohistochemistry (IHC)

IHC was carried out exactly as described [20]. Antibodies for ALAS1 (sc-50531), HCP1 (sc-134997), HRG1 (sc-101957), HO-1 (sc-10789), CYCS (sc-7159), PTGS2 (sc-7951), GLI1 (sc-20687), UQCRC2 (sc-390378), ATP5F1B (sc-33618), CYP1B1 (sc-32882), and MYC (sc-40) were purchased from Santa Cruz Biotechnology. Antibodies for PGRMC1 (#13856), GAPDH (#5174) and ABL1 (#2862) were purchased from Cell Signaling Technology and for Pimonidazole Hydrochloride from Hydroxyprobe, Inc., respectively. Antibodies for NOX4 (ab133303), PDHA1 (ab92696), SLC2A1 (ab40084), and MCL1 (ab32087) were purchased from Abcam. Antibodies for FLNA (sc-28284), SMARCC2 (sc-10757), EFNA2 (sc-631), and HSPB1 (sc-9012) were purchased from Santa Cruz Biotechnology. Antibodies for HIF1α (NB100-123), KDELC1 (NBP1-97469SS) KDM4C (NBP-49600), SLC40A1 (NBP-21502), and TFRC (NB100-92243) were purchased from Novus Biologicals. Antibodies for CA9 (100-417), HRP-conjugated goat anti-mouse IgG (NB7539) and for Hexokinase II (PA5-29326), HRP-conjugated goat anti-rabbit IgG (#31460) were purchased from Novus and Thermo Fisher Scientific, respectively. The detection of DNA fragmentation in tumor tissues was carried out using the TumorTACS^TM^ *In Situ* Apoptosis Detection Kit, following the manufacturer’s protocol.

### IHC imaging and quantification

Slides were scanned at 40X with an Olympus VS120 slide scanner and quantified using cellSens 1.16 software (Olympus), exactly as described^59^. Briefly, multiple regions of interest (ROIs) of equal area were drawn over tumor regions. ROIs were positioned evenly throughout tumor regions and were retested under three different filters—FITC, Cy3, and Cy5—to exclude any artifacts. Mean signal intensity from all ROIs were averaged, and the corresponding negative control average was subtracted to yield the signal intensity for each antigen.

### Statistical analysis

Data from different treatment groups of cells, mice, and tissues were compared, and statistical analyses were performed with a Welch 2-sample t-test. An n of 6 per group will provide enough statistical power to detect a 50% difference with a power of 95% and a p-value of 0.05.

### Ethical approval

Approval: Experimental protocol # 13-05 was approved by IACUC of the University of Texas at Dallas.

Accordance: All experimental methods on animals were carried out in accordance with the relevant guidelines approved under protocol # 13-05.

## Supplementary information

Supplementary figures and legends

## Data Availability

The datasets generated during and/or analyzed during the current study are available from the corresponding author upon request.
