# Supplementary material for: Cyclopamine tartrate, a modulator of hedgehog signaling and mitochondrial respiration, effectively arrests lung tumor growth and progression
Source: Sci Rep. 2019 Feb 5;9:1405. doi: 10.1038/s41598-018-38345-1 (PMC6363760; doi:10.1038/s41598-018-38345-1)
Supplement: Supplementary file 1 — Supplementary figures and legends [file 41598_2018_38345_MOESM1_ESM.pdf]

Cyclopamine tartrate, a modulator of hedgehog signaling and mitochondrial respiration, effectively arrests lung tumor growth and progression

Sarada Preeta Kalainayakan[1], Poorva Ghosh[1], Sanchareeka Dey[1], Keely E Fitzgerald[1], Sagar Sohoni[1], Purna Chaitanya Konduri[1], Massoud Garrossian[2], Li Liu[3]* and Li Zhang[1]*

[1]Department of Biological Sciences, University of Texas at Dallas, Richardson, TX 75080, USA

[2]Logan Natural Products, Plano, TX 75025, USA

[3]Department of Radiology, The University of Texas Southwestern Medical Center, Dallas, TX

*Correspondence:

Li Liu, Ph.D., Department of Radiology, University of Texas Southwestern Medical Center,
5323 Harry Hines Blvd., Dallas, Texas 75390-9058, USA
Phone: 214-648-8059, Fax: 214-648-4538, E-mail: Li.Liu@UTSouthwestern.edu

Li Zhang, Ph.D., Department of Biological Sciences, The University of Texas at Dallas,
800 W Campbell Rd, Mail stop RL11, Richardson, Texas, 75080
Phone: 972-883-5757, Fax: 972-883-5759, E-mail: li.zhang@utdallas.edu

Supplemental figures and legends

Figure S1

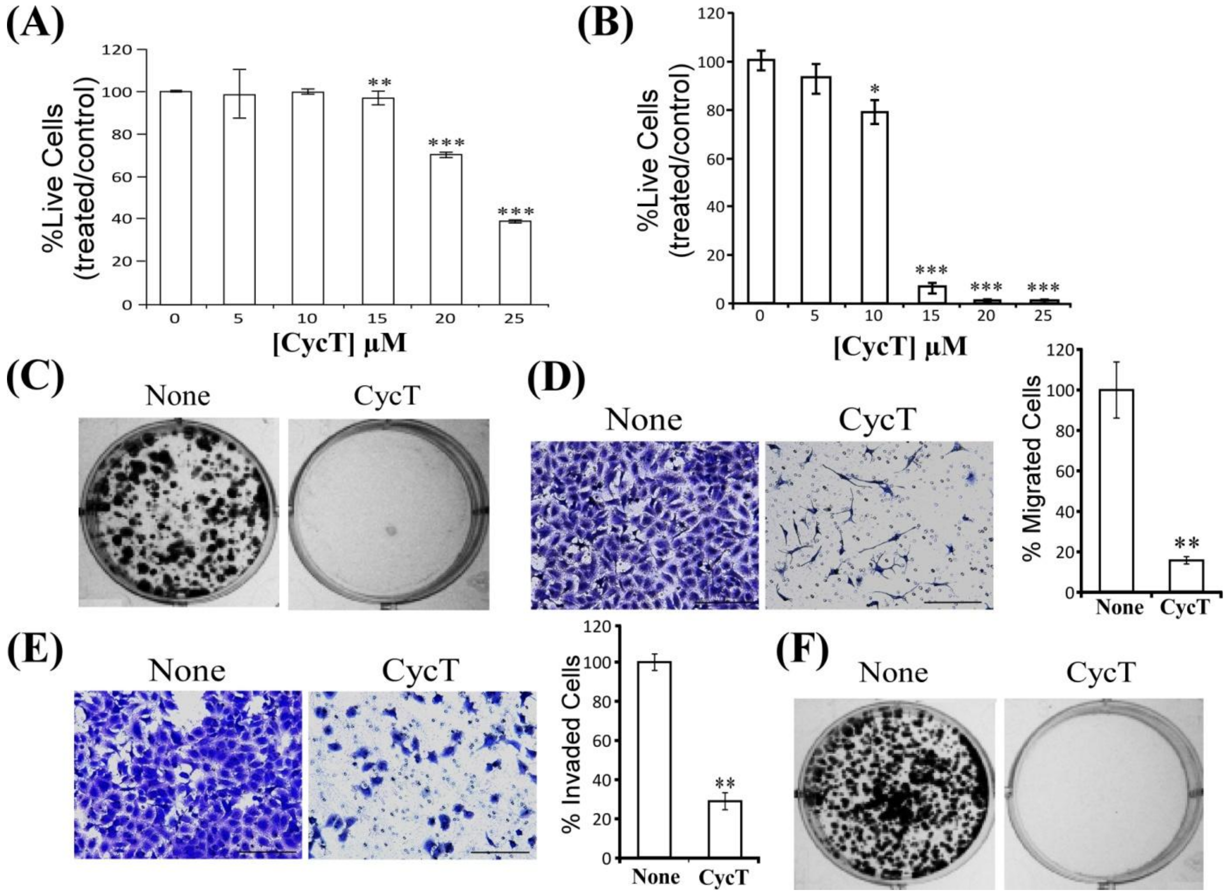

Fig. S1 (A) The dose response of H1299 NSCLC cell proliferation to CycT. (B) The dose response of A549 NSCLC cell proliferation to CycT. (C) CycT inhibits colony formation by H1299 cells. (D) CycT effectively inhibits migration by A549 NSCLC cells. (E) CycT effectively inhibits invasion by A549 NSCLC cells. Scale bar: 200 μm. Data are plotted as mean ± standard deviation. (F) CycT effectively inhibits colony formation by A549 NSCLC cells. For statistical analysis, the levels in treated cells were compared to the levels in untreated cells with a Welch 2-sample t-test. **, p-value < 0.005; ***, p-value < 0.0005.

Figure S2

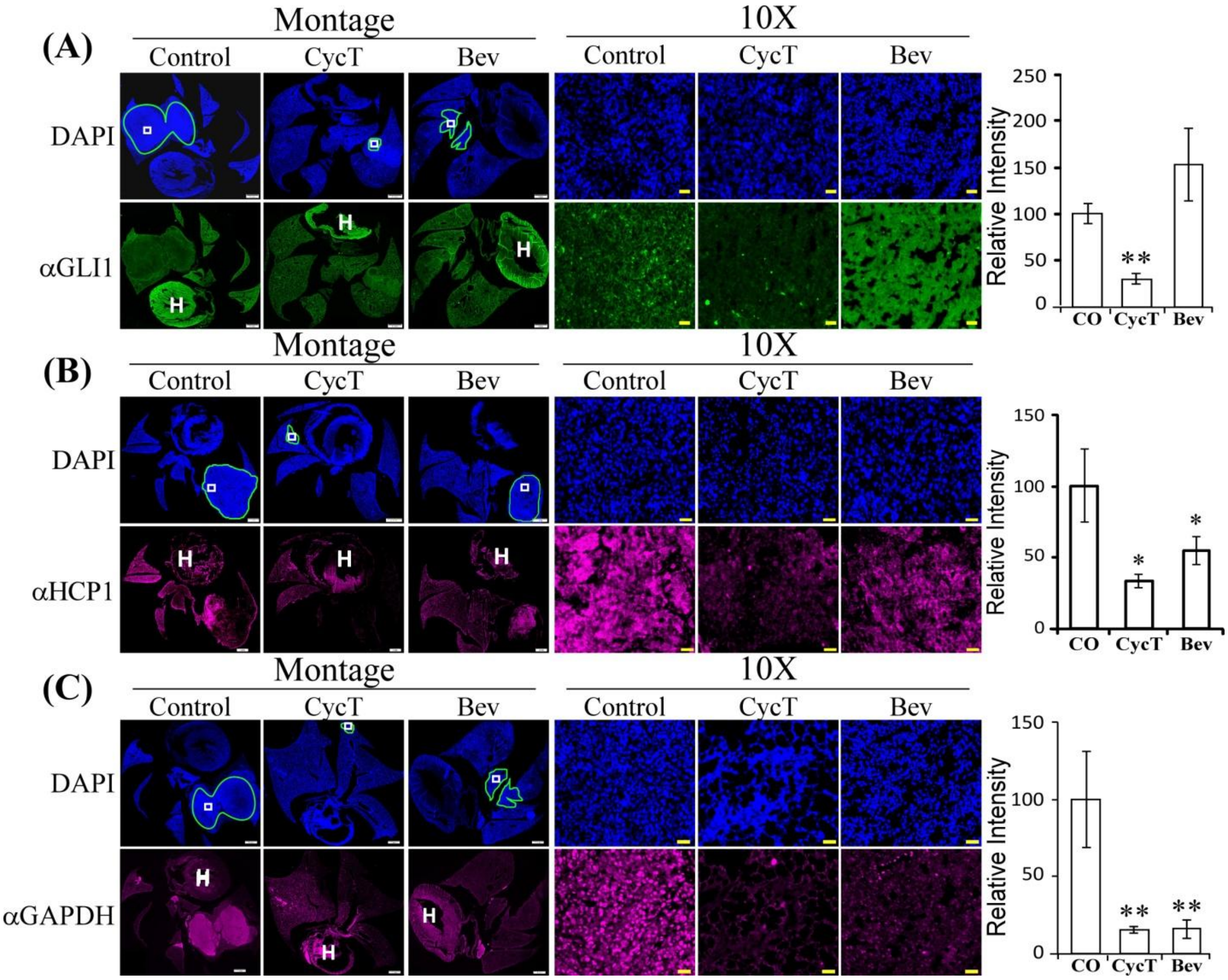

Fig. S2 (A) The effects of CycT and Bev on the levels of the transcriptional regulator GLI1 mediating Hh signaling in orthotopic tumor xenografts. (B) The effects of CycT and Bev on the levels of the heme transporter HCP1 in orthotopic tumor xenografts. (C) The effects of CycT and Bev on the levels of the putative heme-binding and chaperone protein GAPDH in orthotopic tumor xenografts. Scale bar: Montage, 1 mm; 10X, 20 μm. Data are plotted as mean ± SEM. For statistical analysis, the levels in treated tumors were compared to the levels in control tumors with a Welch 2-sample t-test. **, p-value < 0.005. IHC images are representative of 3 independent experiments.

Figure S3

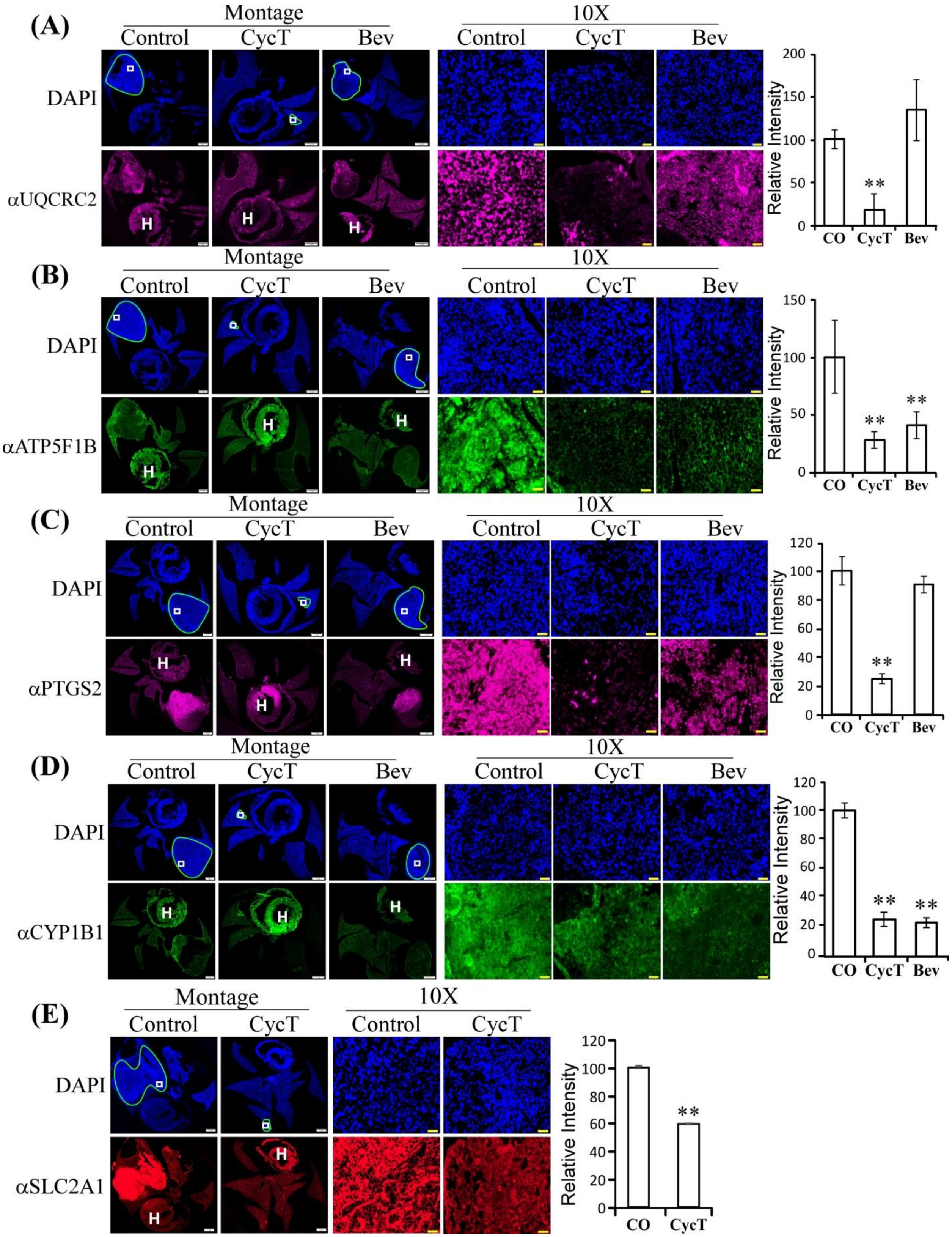

(A)
Montage
Control
CycT
Bev
10X
Control
CycT
Bev
DAPI
αUQCRC2
H
Relative Intensity
200
150
100
50
0
**
CO
CycT
Bev
(B)
Montage
Control
CycT
Bev
10X
Control
CycT
Bev
DAPI
αATP5F1B
H
Relative Intensity
150
100
50
0
**
**
CO
CycT
Bev
(C)
Montage
Control
CycT
Bev
10X
Control
CycT
Bev
DAPI
αPTGS2
H
Relative Intensity
120
100
80
60
40
20
0
**
CO
CycT
Bev
(D)
Montage
Control
CycT
Bev
10X
Control
CycT
Bev
DAPI
αCYP1B1
H
Relative Intensity
120
100
80
60
40
20
0
**
**
CO
CycT
Bev
(E)
Montage
Control
CycT
10X
Control
CycT
DAPI
αSLC2A1
H
Relative Intensity
120
100
80
60
40
20
0
**
CO
CycT

Fig. S3 The effects of CycT and Bev on the levels of UQCRC2 (A), ATP5F1B (B), PTGS2 (C), and CYP1B1 (D) in orthotopic tumor xenografts. (E) The effects of CycT on the levels of SLC2A1 in orthotopic tumor xenografts. Scale bar: Montage, 1 mm; 10X, 20 μm. Data are plotted as mean ± SEM. For statistical analysis, the levels in treated tumors were compared to the levels in control tumors with a Welch 2-sample t-test. **, p-value < 0.005. IHC images are representative of 3 independent experiments.

Figure S4

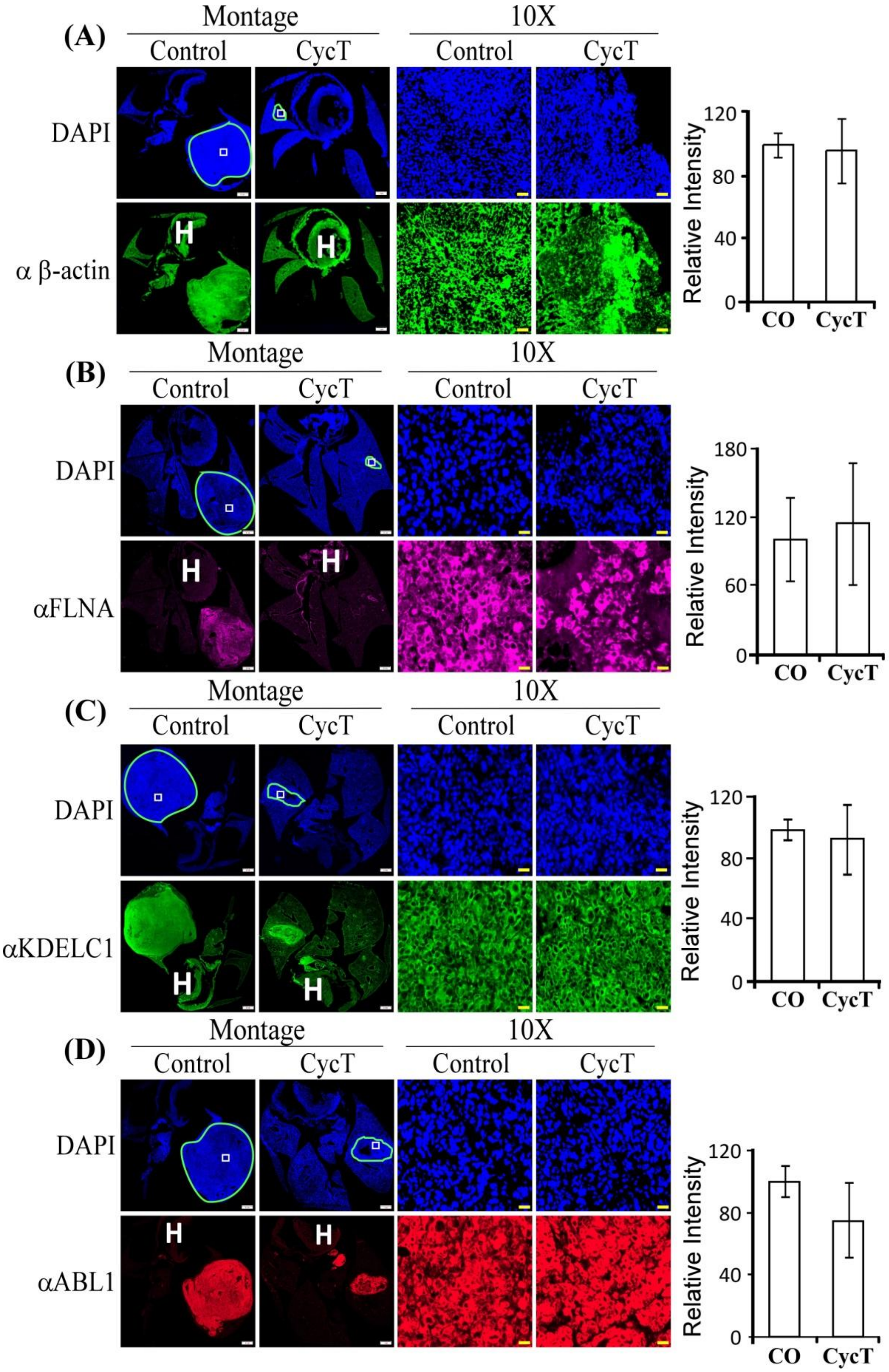
(A)
Montage
10X
Control
CycT
Control
CycT
DAPI
α β-actin
H
H
120
80
40
0
Relative Intensity
CO
CycT
(B)
Montage
10X
Control
CycT
Control
CycT
DAPI
αFLNA
H
H
180
120
60
0
Relative Intensity
CO
CycT
(C)
Montage
10X
Control
CycT
Control
CycT
DAPI
αKDELC1
H
H
120
80
40
0
Relative Intensity
CO
CycT
(D)
Montage
10X
Control
CycT
Control
CycT
DAPI
αABL1
H
H
120
80
40
0
Relative Intensity
CO
CycT

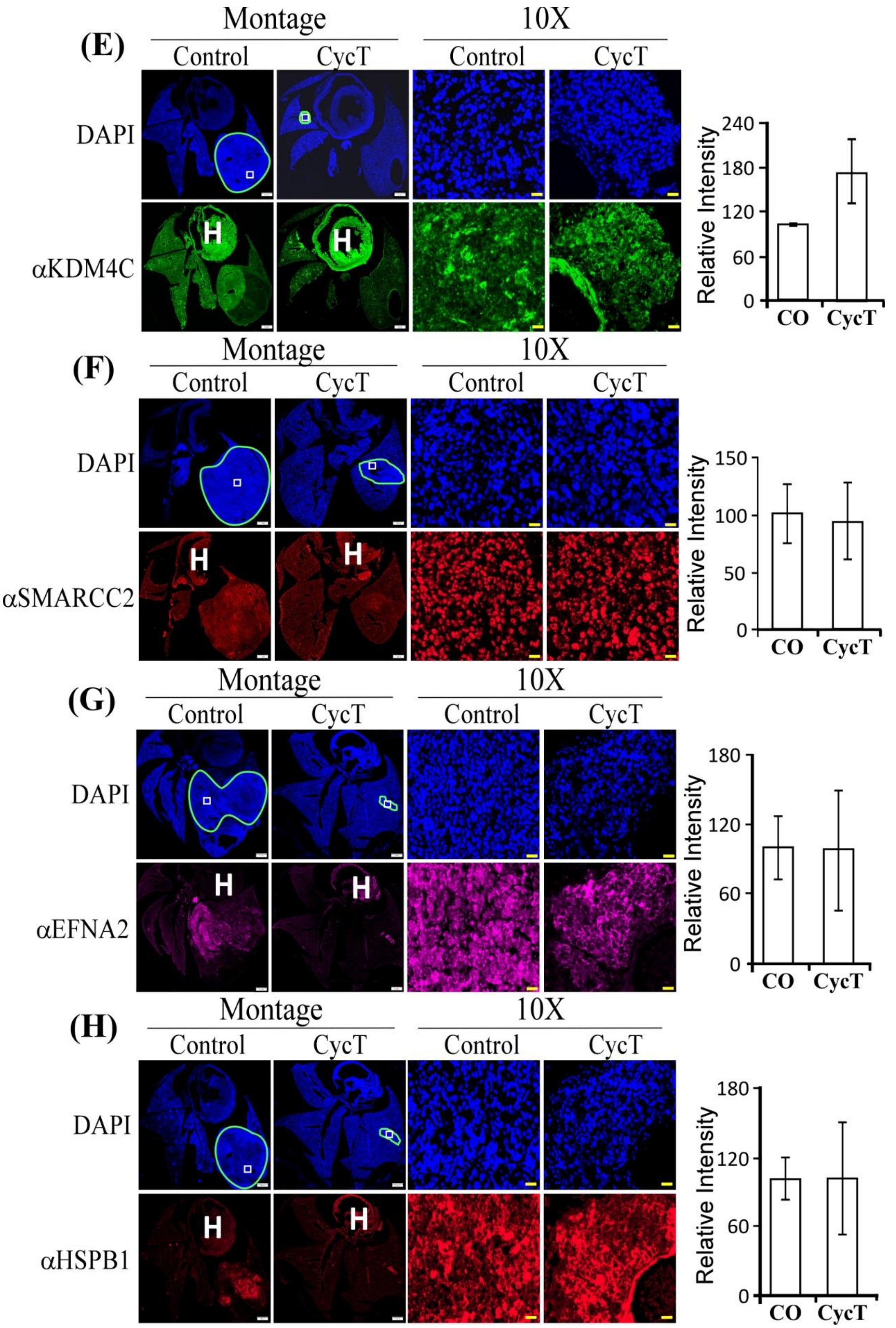

(E)
Montage
10X
Control
CycT
Control
CycT
DAPI
αKDM4C
H
Relative Intensity
240
180
120
60
0
CO
CycT
(F)
Montage
10X
Control
CycT
Control
CycT
DAPI
αSMARCC2
H
Relative Intensity
150
100
50
0
CO
CycT
(G)
Montage
10X
Control
CycT
Control
CycT
DAPI
αEFNA2
H
Relative Intensity
180
120
60
0
CO
CycT
(H)
Montage
10X
Control
CycT
Control
CycT
DAPI
αHSPB1
H
Relative Intensity
180
120
60
0
CO
CycT

Fig. S4 CycT did not affect the levels of an array of structural and signaling proteins β-actin (A), filamin FLNA (B), ER marker KDELC1 (C), signaling and transcriptional proteins ABL1 (D), KDM4C (E), SMARCC2 (F), a lymphoid-specific member of the Ets family EFNA2 (G), and heat shock protein Hsp27 (HSPB1) (H). Scale bar: Montage, 1 mm; 10X, 20 μm. Data are plotted as mean ± SEM. For statistical analysis, the levels in treated tumors were compared to the levels in control tumors with a Welch 2-sample t-test. IHC images are representative of 3 independent experiments.

Figure S5

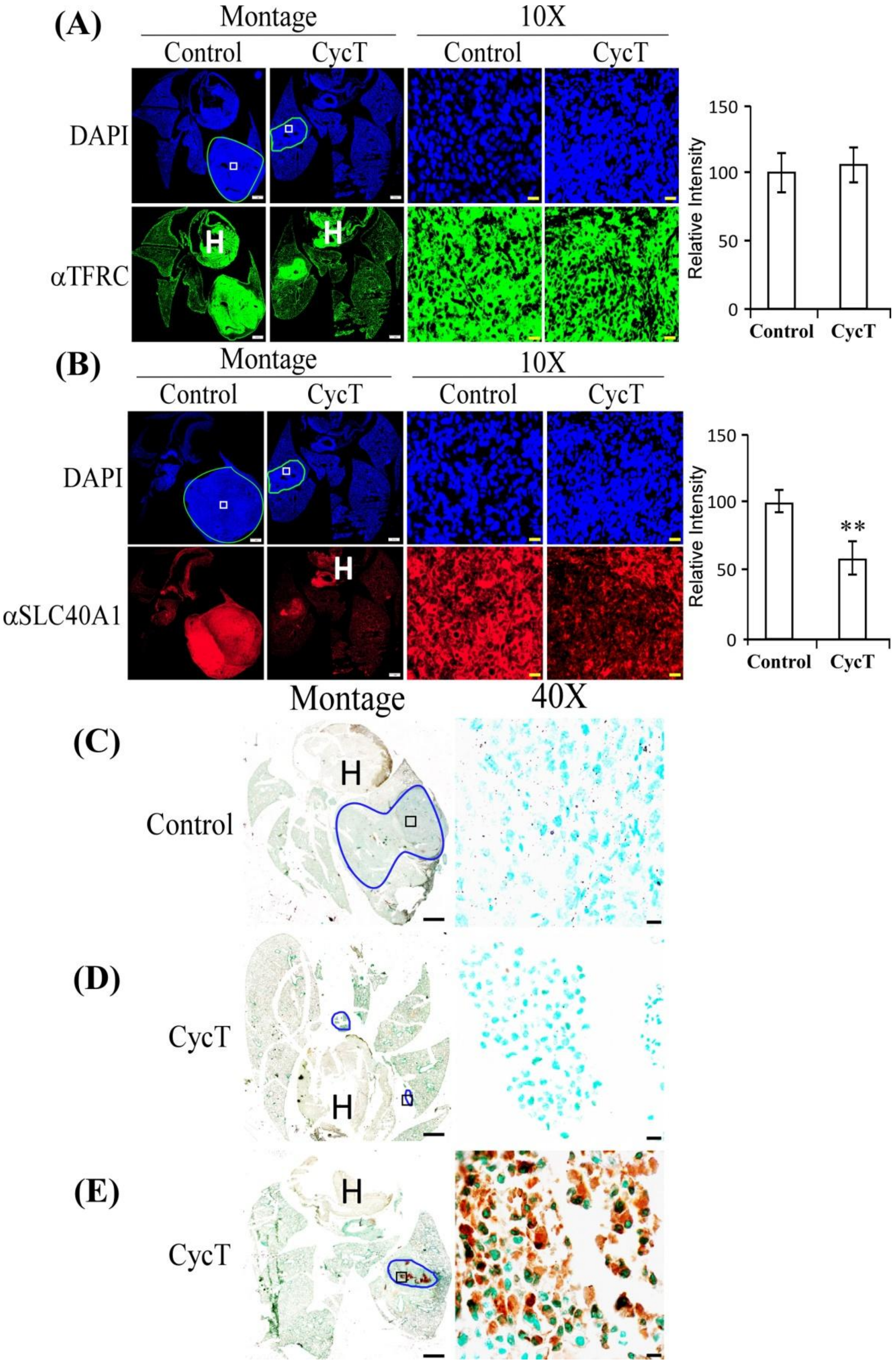

Fig. S5 (A) The effects of CycT the levels of proteins involved in cellular iron uptake transferrin receptor TFRC (B) and iron exporter, ferroportin (SLC40A1) (B). Scale bar: Montage, 1 mm;

10X, 20 μm. Data are plotted as mean ± standard deviation. For statistical analysis, the levels in treated cells were compared to the levels in untreated cells with a Welch 2-sample t-test. **, p-value < 0.005. (C) The *in situ* apoptosis detection assay did not detect signs of DNA fragmentation in control tumors without CycT treatment. (D) The *in situ* apoptosis detection assay detected no signs of DNA fragmentation in CycT-treated tumors with a small size. (E) The *in situ* apoptosis detection assay detected signs of DNA fragmentation in CycT-treated tumors with a slightly larger size. Tumor tissues are from orthotopically implanted lung tumors as shown in Fig. 3.
